# PACAP activates MRGPRX2 on meningeal mast cells to drive migraine-like pain

**DOI:** 10.1038/s41598-023-39571-y

**Published:** 2023-07-29

**Authors:** Sami Sbei, Taylor Moncrief, Nathachit Limjunyawong, Yaping Zeng, Dustin P. Green

**Affiliations:** 1 Institute for Translational Sciences, University of Texas Medical Branch, Galveston, TX USA; 2 Department of Neurobiology, University of Texas Medical Branch, Galveston, TX USA; 3 Center of Research Excellence in Allergy and Immunology, Research Department, Faculty of Medicine Siriraj Hospital, Mahidol University, Bangkok, 10700 Thailand

**Keywords:** Innate immune cells, Neuroimmunology, Pain

## Abstract

Migraine ranks among the most prevalent disorders worldwide, leading to disability and decreased quality of life in patients. Recently, neurogenic inflammation has been recognized as a potential underlying pathology contributing to the migraine pain pathway. Mast cells reside in the meninges and have been implicated in contributing to the pathophysiology of migraine. Here we report for the first time that the mouse Mas-Related G-protein-coupled Receptor B2 (MrgprB2), is expressed on meningeal connective tissue mast cells and contributes to Pituitary Adenylate Cyclase Activating Peptide (PACAP)-induced migraine-like pain behavior. We also found that PACAP was able to dose-dependently lead to enzyme release from human mast cells via activation of MRGPRX2; the human homolog of MrgprB2. Using a transgenic MRGPRX2 mouse, we observed significant increases in PACAP-induced migraine-like pain behavior in MRGPRX2^+^ mice vs mice lacking the receptor. These results reveal both MrgprB2 and MRGPRX2 as important contributors to neuropeptide-induced migraine pain.

## Introduction

Migraine is a chronic episodic headache disorder and is one of the top five most prevalent disorders worldwide. Migraines are considered among the leading causes of disability, as severe pain is worsened by physical activity and is accompanied by central nervous system (CNS) sensory and/or motor dysfunction in up to 25% of patients^1^. Despite the high prevalence of migraine and the severity at which it affects patients’ quality of life, gaps remain in understanding the underlying pathophysiology. Among the mechanisms attributed to causing migraine pain, recently, the neurogenic inflammation pathway has gained more recognition. The link between immune cells mounting a localized inflammatory response is proposed to be involved in the migraine pain pathway^1,2^.

Mast cells are tissue-resident innate immune cells that play an important role in pathogen clearance, immune-cell recruitment, and inflammatory-mediated pain^3,4^. Mast cells reside in the meninges, the protective connective tissue surrounding the brain, and are of special interest for research concerning headaches and migraine pain^5,6^. Meningeal mast cells are found in close proximity to nerve fibers and are shown to degranulate in response to nociceptor activation, contributing to the migraine pain pathway^7,8^. Importantly, in human studies, a clinical relationship has been established between mast cell activation and primary headache syndromes^9^. However, specific mast cell receptor subtypes and underlying cellular and molecular mechanisms that promote mast cell activation during migraine have yet to be elucidated.

PACAP is a vasoactive neuropeptide found in the trigeminovascular system, where it was shown to be elevated in the acute phase of migraine^10^. Intravenous infusion of PACAP can evoke migraine-like pain in non-migraineurs similar to how it evokes migraine in patients who suffer from episodic migraine^11^, and is elevated during the ictal phase^10^. Additionally, clinical trials are now underway targeting the PACAP1-38 receptor, Pituitary adenylate cyclase-activating polypeptide type I receptor (PAC1R), for the prophylactic treatment of migraine. However, one study found that targeting PAC1R had no benefit over placebo for migraine prevention^12^, suggesting an alternative receptor pathway may be involved in mediating PACAP-induced migraine. Also, a recent clinical study using a humanized monoclonal antibody directed against PACAP1-38 was successful in reducing headaches, indicating that targeting the peptide may still be an effective migraine treatment^13^. PACAP1-38 was shown to induce headache-like pain via mechanisms independent of CGRP, further highlighting how PACAP1-38 is a separate and distinct mechanism that underlies migraine^14,15^.

In our previous study, we found the mast cell-specific receptor MrgprB2, and its human homolog MRGPRX2^16^, to contribute to neuropeptide-evoked pain^3^. Furthermore, studies examining the rat Mrgpr receptor MrgprB3, thought to be the homolog to MRGPRX2, found PACAP to potently degranulate rat meningeal mast cells via MrgprB3^17^. Given our findings and the implication of mast cells in migraine pain, we sought to examine meningeal MrgprB2^+^ mast cells and whether a similar trend of PACAP-dependent MrgprB2 activation and involvement in pain can be observed in the meninges. The present study demonstrates the presence of MrgprB2^+^ mast cells in meningeal tissue while showing that MrgprB2 activation plays a role in migraine-like pain in vivo. Furthermore, using a newly generated transgenic mouse line, we show that MRGPRX2 is potently activated by PACAP and leads to migraine-like pain.

## Results

### The mast cell receptor MrgprB2 is expressed in the meninges and its activation leads to migraine-like pain behavior

**Figure 1:**
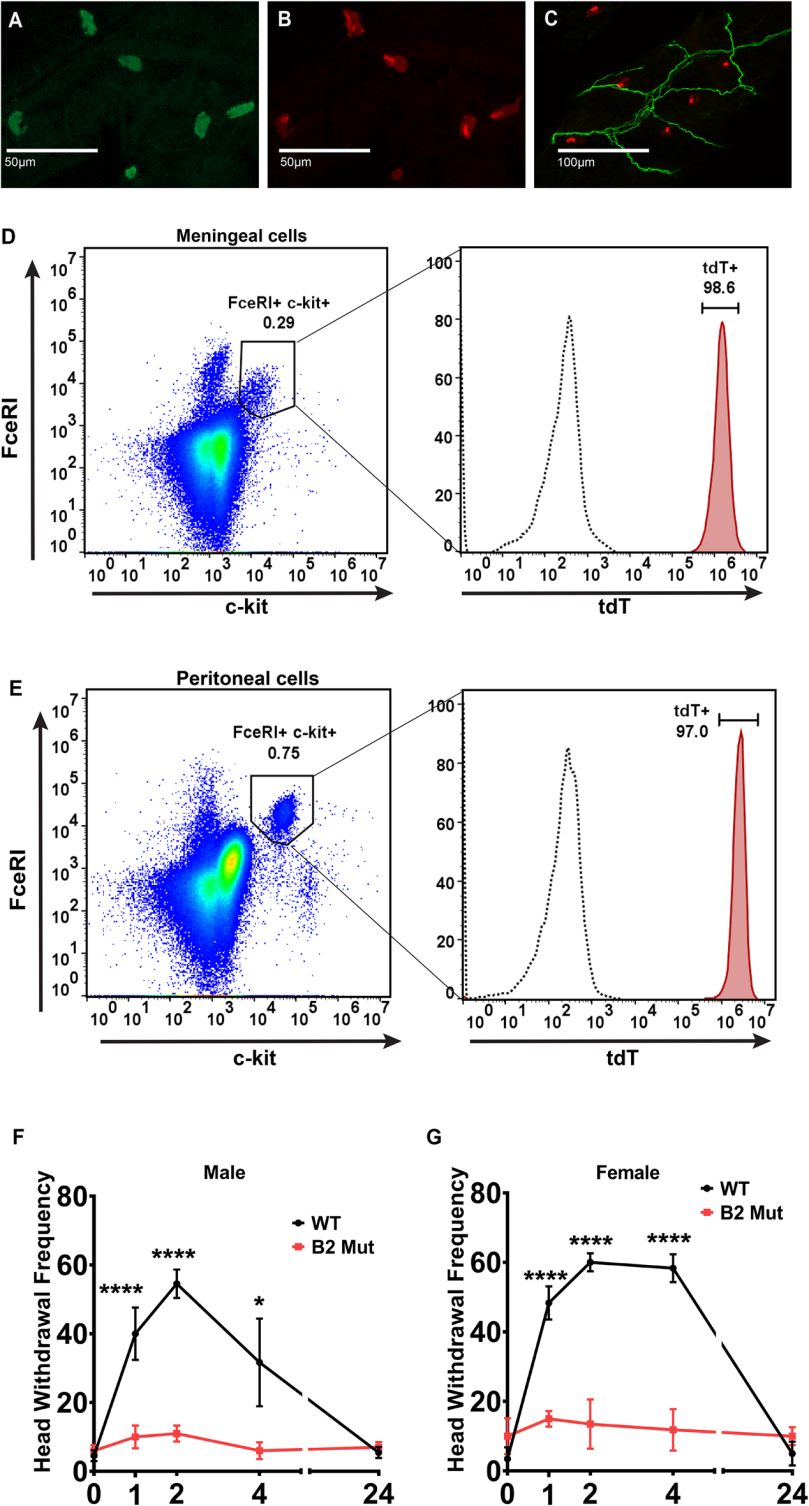
MrgprB2 is expressed on meningeal mast cells and contributes to mechanical hypersensitivity in a mouse model of migraine-like pain. (**A–C**) Confocal images from transgenic mouse meninges in which the tdTomato (tdT) fluorescent protein is under the MrgprB2 promoter (MrgprB2 Cre tdT). (**A**) Avidin staining was used to identify mast cells (green). (**B**) MrgprB2 Cre tdT^+^ mast cells (red). The percentage of tdT^+^ cells that also were avidin-positive was 96%. Over 200 cells were counted from n = 3 mice. (**C**) MrgprB2 Cre tdT^+^ mast cells (red) shown near meningeal afferents (stained with Neurofilament 200, green). The scale bar is 50 μm in (**A,B**) and 100 μm in (**C**). (**D,E**) Representative flow cytometric profile of MrgprB2 Cre MRGPRXB2^+^ tdT^+^ (tdT^+^) mast cells. Cells were harvested from the meninges (**D**) or peritoneal cavity (**E**) and mast cells were identified as live c-kit^+^ FcεRI^+^ cells. An n = 3 of mice per group for both meningeal and peritoneal extracts. (**F,G**) A non-invasive dural stimulation model was used to apply Compound 48/80 (1 mg/kg) to the WT and MrgprB2^−/−^ (mut) mouse meninges. (**F**) Mechanical facial hypersensitivity was measured in male mice prior to dural injection and then 1 h, 2 h, 4 h, and 24 h after dural stimulation with Compound 48/80; WT n = 11, MrgprB2^−/−^ n = 10. (**G**) Female mice were also tested for mechanical hypersensitivity at 1 h, 2 h, 4 h, and 24 h after dural stimulation with Compound 48/80; WT n = 6, MrgprB2^−/−^ n = 6. Error bar: S.E.M. *p < 0.05, ****p < 0.001.

Previous studies have shown that MrgprB2 expression was highly specific to connective tissue mast cells^3,4,16,18^. Moreover, the MrgprB3 which is thought to be the rat homolog of mouse MrgprB2 and human MRGPRX2 is expressed in connective tissue mast cells including the meninges^17^. To confirm the expression of MrgprB2 on mouse meningeal mast cells we began by performing immunohistochemistry staining on mouse meningeal tissue. We used avidin staining to identify mast cells in the meninges of mice expressing the tdTomato fluorescent protein under the MrgprB2 promoter (MrgprB2 Cre tdT) (Fig. 1A, B). B2 Cre expression has been shown to be highly specific to connective tissue mast cells, where double staining with avidin has shown B2 Cre tdT^+^ mast cells at percentages as high as 99.2% in connective tissue^16^. Similar to the level of expression that has been previously reported, we found that 97% of avidin-positive meningeal mast cells were also tdT^+^ and that 96% of tdT^+^ cells were also avidin-positive. We had previously found MrgprB2^+^ mast cells in close proximity to peripheral nerve endings^3^. Comparable to those findings, MrgprB2^+^ mast cells were found near peripheral nerve endings in the meninges as well (Fig. 1C). Furthermore, flow cytometric analysis of MrgprB2 Cre tdT mast cells revealed that the majority of c-kit^+^ FcεRI^+^ (gated from live CD45^+^) cells were also MrgprB2-expressing mast cells (Fig. 1D). We observed a similar expression pattern of MrgprB2 Cre tdT^+^ connective tissue mast cells taken from the peritoneal cavity (Fig. 1E).

To further confirm the role of MrgprB2 expression in the meninges and evaluate this mast cell-specific receptor’s role in mediating migraine pain we utilized the minimally invasive dural stimulation migraine model^19^. This preclinical model allows for a simple minimally invasive application of drugs to the dura mater. Compound 48/80 is a potent mast cell degranulator and works via activation of MrgprB2 in mice and MRGPRX2 in humans^16,20^. Sub-anaphylactic doses of 48/80 have been utilized previously as a model of migraine used to test both withdrawal behavior and meningeal afferent activation^8,21^. We applied Compound 48/80 (1 mg/kg) onto the dura and compared Wild type (WT) to *Mrgprb2*^−/−^ mice (Fig. 1F, G). We observed a significant increase in mechanical facial hypersensitivity in WT animals compared to baseline. However, compared to WT, *Mrgprb2*^−/−^ male and female mice had significant reductions in mechanical facial hypersensitivity. Although MrgprB2 has been confirmed to be expressed on connective tissue mast cells, here we show for the first time evidence for its expression in the meninges and its role in mediating migraine-like pain.

### The neuropeptide PACAP1-38 activates MrgprB2 to stimulate mast cell release and migraine-like pain

**Figure 2:**
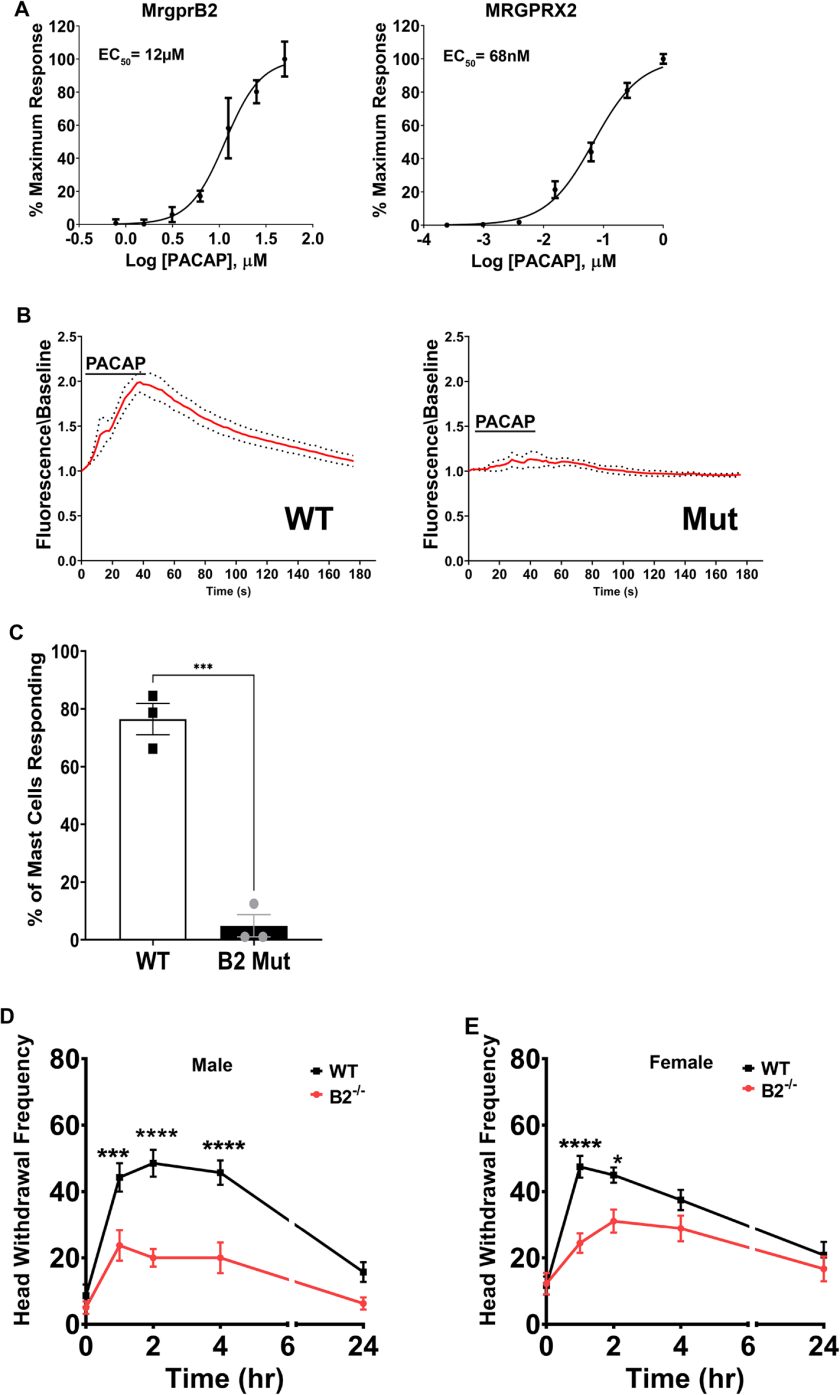
The neuropeptide PACAP1-38 activates MrgprB2 and increases migraine-like pain behavior in MrgprB2, but not MrgprB2^−/−^ mice. (**A**) Dose–response curve of PACAP1-38 on activation of HEK293 cells expressing MrgprB2 and MRGPRX2. (**B**) Average change in intracellular calcium (red line) in peritoneal mast cells from WT or MrgprB2^−/−^ (mut) mice activated by PACAP1-38 (10 μM), with ± 95% confidence intervals shown (black points), n = 3 mice per group. (**C**) The percentage of WT and MrgprB2^−/−^ (mut) peritoneal mast cells responding to PACAP1-38. Two-tailed unpaired Student’s t-test, ***p < 0.001, n = 3 mice per group. (**D,E**) The non-invasive dural stimulation model was used to apply PACAP1-38 (10 μM) to the WT and Mrgprb2^−/−^ (mut) mouse meninges. (**D**) Mechanical hypersensitivity was measured in male mice prior to dural injection and then 1 h, 2 h, 4 h, and 24 h after dural stimulation with PACAP1-38; WT n = 7, MrgprB2^−/−^ (mut) n = 8. (**E**) Female mice were also tested for mechanical hypersensitivity at 1 h, 2 h, 4 h, and 24 h after dural stimulation with PACAP1-38; WT n = 11, MrgprB2^−/−^ n = 9. Error bar: S.E.M. *p < 0.05, ****p < 0.001.

Because neuropeptides were previously shown to activate MrgprB2^3^ along with our findings that MrgprB2^+^ mast cells are abundant in the meninges, we next investigated the migraine-inducing neuropeptide PACAP1-38 for its possible role in MrgprB2 activation. Utilizing HEK293 cells expressing MrgprB2, we found that PACAP1-38 activated MrgprB2 (EC_50_ of 11.54 ± 0.05 μM) and MRGPRX2 (EC_50_ of 68.48 ± 0.04 nM) (Fig. 2A). We next moved to in vitro functional assays, where we found that PACAP1-38 (10 μM) activated a significant percentage of connective tissue mast cells harvested from the WT mice, but this activation was completely absent in *Mrgprb2*^−/−^ mice (Fig. 2B, C). Dural application of PACAP1-38 (10 μM) was also able to significantly increase mechanical facial hypersensitivity in WT animals compared to baseline (Figs. 2D, E). However, compared to WT, *Mrgprb2*^−/−^ male and female mice both had significant reductions in mechanical facial hypersensitivity. Taken together, these findings reveal the migraine-inducing peptide PACAP1-38 is an agonist for MrgprB2, capable of inducing MrgprB2-mediated migraine-like pain.

### PACAP1-38 activates mouse meningeal mast cells that express the human receptor MRGPRX2

**Figure 3:**
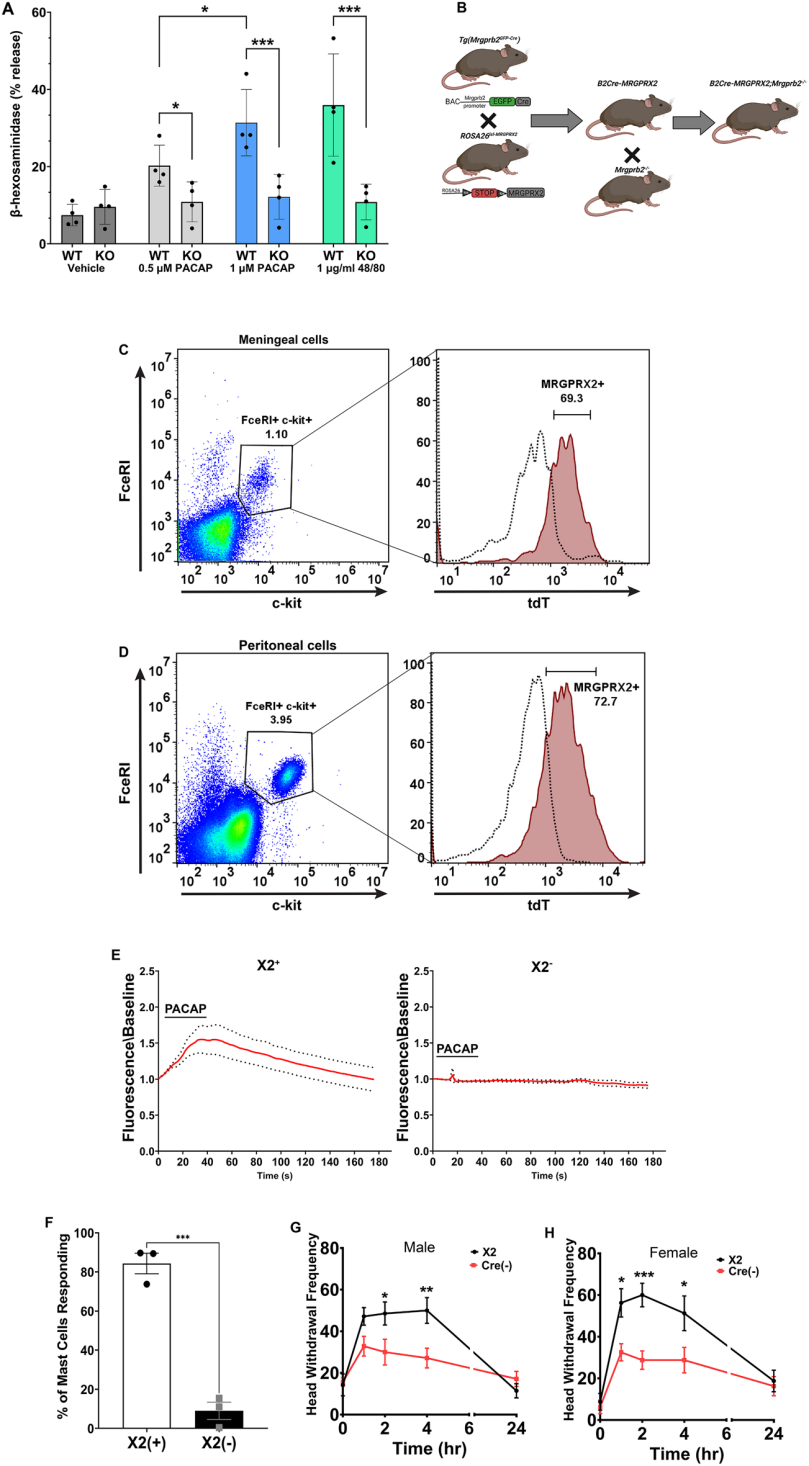
PACAP1-38 increases migraine-like pain behavior in mice with mast cells expressing the human receptor MRGPRX2. (**A**) PACAP1-38 dose-dependent induced release of β-hexosaminidase from WT LAD2 human mast cells compared to LAD2 lacking MRGPRX2. N = 3, Error bar: S.E.M. *p < 0.05, ****p < 0.001. (**B**) Diagram showing the mating strategy to generate humanized (X2^+/+^) mice. (**C,D**) Representative flow cytometric profile of MrgprB2 Cre MRGPRX2^+^ (X2^+^) mast cells. Cells were harvested from the meninges (**C**) or peritoneal cavity (**D**) and mast cells were identified as live c-kit^+^ FcεRI^+^ cells. An n = 3 of mice per group for both meningeal and peritoneal extracts. (**E**) Average change in intracellular calcium (red line) in peritoneal mast cells from MrgprB2 Cre^+^ MRGPRX2^+^ (X2^+^) or MrgprB2 Cre^−^ MRGPRX2^+^ (X2^−^) mice activated by PACAP1-38 (1 μM), with ± 95% confidence intervals shown (black points), n = 3 per group. (**F**) The percentage of MrgprB2 Cre^+^ MRGPRX2^+^ (X2^+^) or MrgprB2 Cre^−^ MRGPRX2^+^ (X2^−^) peritoneal mast cells responding to PACAP1-38. Two-tailed unpaired Student’s t-test, ***p < 0.001, n = 3 mice per group. (**G,H**) The non-invasive dural stimulation model was used to apply PACAP1-38 (1 μM) to the MrgprB2 Cre^+^ MRGPRX2^+^ (X2^+^) or MrgprB2 Cre^−^ X2^+^ (X2^−^) mouse meninges. (**G**) Mechanical hypersensitivity was measured in male mice prior to dural injection and then 1 h, 2 h, 4 h, and 24 h after dural stimulation with PACAP1-38; X2^+^ n = 7, X2^−^ n = 7. (**H**) Female mice were also tested for mechanical hypersensitivity at 1 h, 2 h, 4 h, and 24 h after dural stimulation with PACAP1-38; X2^+^ n = 8, X2^−^ n = 8. Error bar: S.E.M. *p < 0.05, ***p < 0.001 comparing X2^+^ to X2^−^ mice. Diagram created with BioRender.

To assess the activation of human MRGPRX2 by PACAP1-38, we used Laboratory of Allergic Diseases 2 (LAD2) cells, an immortalized human mast cell line that expresses MRGPRX2. Application of PACAP1-38 was capable of dose-dependently inducing the release of β-hexosaminidase -one of the enzymatic components of the mast cell granules- from WT LAD2 cells but not LAD2 cells in which MRGPRX2 had been knocked out (Fig. 3A).

To test PACAP1-38 activation of MRGPRX2 in vivo we used a transgenic mouse line that expresses MRGPRX2 in mouse mast cells (X2^+^ mice) lacking MrgprB2 (Fig. 3B). Mice lacking MrgprB2 Cre will not express MRGPRX2 and were used as a control (X2^−^). We next used flow cytometry to examine the expression of MRGPRX2 in both the meningeal and peritoneal mast cells (Fig. 3C, D). Similar to the results observed with MrgprB2-expressing mice, flow cytometric analysis of X2^+^ mouse mast cells found that the majority of c-kit^+^ FcεRI^+^ (gated from live CD45^+^) cells were also MRGPRX2-expressing mast cells.

To test whether PACAP1-38 was able to activate X2^+^ mast cells we used calcium imaging. A significant percentage of peritoneal mast cells harvested from X2^+^ mice were activated by PACAP1-38 (1 μM) in contrast to X2^−^ mice (Fig. 3E, F). Lastly, we examined if PACAP1-38 (1 μM) could evoke mechanical facial hypersensitivity in our X2^+^ mice. Dural application of PACAP1-38 was able to significantly increase mechanical facial hypersensitivity in X2^+^ animals compared to X2^−^ mice. However, X2^−^ mice had significant reductions in mechanical facial hypersensitivity (Fig. 3G, H) compared to X2^+^ animals. The data presented here demonstrate, for the first time, a mouse line that functionally expresses MRGPRX2 in connective tissue mast cells, including meningeal mast cells, and responds to PACAP1-38 to contribute to migraine-like pain.

## Discussion

Mast cells have long been linked to migraine^5,7,8^, but the specific mast cell receptor subtypes involved were only presumed. Here we identify the mast cell-specific receptor, MrgprB2, and its human homolog, MRGPRX2, in mediating PACAP1-38-induced migraine-like pain. Our previous work showed for the first time that MrgprB2-positive mast cells are abundant in the skin where they are localized with peripheral nerve terminals^3^. Although mast cells are found in the meninges, as others and our data confirm, there has been no study of the localization of MrgprB2 in meningeal mast cells. Here we present data showing an almost complete overlap of MrgprB2 Cre tdT^+^ cells with the mast cell marker avidin. Moreover, these MrgprB2 Cre tdT^+^ mast cells were found in close proximity to meningeal afferent fibers. Using a preclinical model of migraine-like pain behavior, we found activation of MrgprB2^+^ meningeal mast cells by Compound 48/80 could evoke facial mechanical hypersensitivity in mice.

Compound 48/80 provides compelling evidence for implicating MrgprB2 activation in contributing to migraine-like pain, but we next wanted to explore what endogenous agonist might play a role in activating MrgprB2. The neuropeptide PACAP1-38 is an ideal candidate as it has previously been shown to activate what is thought to be the rat homolog of MRGPRX2^17,22^. Our in vitro dose–response data showed PACAP1-38 was capable of activating MrgprB2 in the low micromolar range and MRGPRX2 in the low nanomolar range. This difference in agonist efficacy, with MrgprB2 having higher EC_50s_ compared to MRGPRX2, has been reported for multiple agonists. These include Compound 48/80 (4 μg/mL vs 470 ng/mL) and endogenous agonists such as Substance-P (54 µM vs 152 nM) and pro-adrenomedullin peptide 9–20 (12 µM vs 166 nM)^16^. Our functional studies showed that PACAP1-38 was capable of activating MrgprB2^+^ connective tissue mast cells ex vivo as well as degranulate MRGPRX2^+^ mast cells. Lastly, PACAP1-38 application to the dura led to increases in mechanical hypersensitivity, which was significantly less in MrgprB2^−/−^ mice. Importantly the time course for PACAP1-38-induced hypersensitivity closely mimicked that seen in clinical studies of PACAP1-38-induced migraine, where most non-migraineur patients reported migraine-like pain within 2 h post-PACAP1-38 administration^11^.

Identification of MrgprB2 as the mouse homolog of human MRGPRX2 has been invaluable to exploring mast cell physiology. However, species differences remain particularly diverse with respect to ligand efficacy. Using this unique mouse line, we found PACAP1-38 was capable of activating X2^+^ mouse mast cells at a dose tenfold lower than MrgprB2^+^ mouse mast cells. Furthermore, the equivalent dose of PACAP1-38 that was able to induce significant mechanical facial hypersensitivity in WT mice led to a 50% increase when applied to MRGPRX2^+^ mouse mast cells.

Interestingly our data show that loss of MrgprB2 significantly attenuated but did not completely abolish PACAP1-38 mediated migraine-like pain compared to WT, possibly pointing to other receptor subtypes being involved. Along with MrgprB2 and MRGPRX2, multiple other receptors can also be activated by PACAP1-38, namely PAC1R and Vasoactive Intestinal Receptors 1 and 2 (VPAC1 and VPAC2)^23^. Antagonists for PAC1R had no effect on preventing PACAP1-38 activation of mast cells^22^ and PACAP1-38’s affinity for VPAC1 and VPAC2 was found to be in the low micro-molar range, similar to what we observed for MrgprB2^24^. Future studies will need to examine if these receptors are expressed on meningeal mast cells and if they too also contribute to PACAP1-38-induced mast cell activation and migraine-like pain. Although this study focused on comparisons of WT to receptor knockout, one caveat of the behavioral results is the lack of vehicle control groups, making it difficult to evaluate the degree to which each group differs from the baseline.

In summary, our findings show that MrgprB2 plays an important role in the migraine pain pathway. Its activation by PACAP1-38 provides an alternative target in the treatment of debilitating migraine and gives further credence to the role of the innate immune system in modulating the peripheral nervous system.

## Methods

### Behavioral assays

Dural injections: non-invasive dural stimulation in mice was performed under light isoflurane anesthesia administered via nose cone (4–5% for induction; 1–2% for maintenance) from a vaporizer. The surgical plane of anesthesia was confirmed with a tail pinch. Acute stimulation of the dura in mice was accomplished using a modified mouse dural injection technique that has been previously described^19^, whereby a cannula injector is placed posterior to the bregma at the sagittal and lambdoidal suture junction. The total volume injected did not exceed 5 µL. The mice were then returned to their home cage to recover and then withdrawal thresholds are tested using Von Frey filaments.

Von Frey methods: mice were acclimated to small cups for at least two hours for three days prior to the first baseline measurement. Facial mechanical hypersensitivity was measured using the Von Frey frequency method. A Von Frey monofilament (0.07 g) was applied to the midline of the skull until the fiber bent, for approximately 1 s. This stimulation was done ten times at 2-s intervals. A positive response was considered if the mouse rapidly withdrew from the filament. Wiping with both paws was considered a grooming response and not counted. If a response was evoked by the applied fiber, mice were allowed to settle before applying the next stimulus. The facial withdrawal due to applied fiber in these 10 trials was expressed as a percent response frequency. Behavioral assays were done with blinded observers.

### Immunohistochemistry staining

Adult mice up to 6 months of age were anesthetized via inhalation anesthesia with vaporized isoflurane (4% for 5 min, or until mice are nonresponsive to toe-pinch) and then perfused with 30 mL 0.1 M phosphate-buffered saline (PBS, pH 7.4, 4 °C) followed with 30 mL of fixative [4% paraformaldehyde (vol/vol), 4 °C]. Tissues were post-fixed in paraformaldehyde at 4 °C for 2 h. Tissues were cryoprotected in 20% sucrose (wt/vol) for up to 8 h followed by 30% sucrose for 24 h and then sectioned (10 μm width) with a cryostat. The sections on slides were dried at 37 °C for 30 min and fixed with 4% paraformaldehyde at room temperature (RT) for 10 min. The slides were pre-incubated in blocking solution (10% normal goat serum (vol/vol, Life Technologies), 0.03% Triton X-100 (vol/vol, Sigma) in PBS (pH 7.4) for 1 h at RT. For avidin staining of mast cells, sections were incubated with avidin sulforhodamine 101 conjugate (Marker Gene Technologies, 1:500 dilution, M1124). The sections were washed three times with PBS and Fluoromount (Southern Biotech) was applied before coverslips were placed over the section.

### Flow cytometry

Meningeal tissue was carefully harvested from the skullcaps as previously described^25^ using an 8 mm biopsy punch (Integra) and extracted by gently scraping the tissue from the bone. Tissue was placed in cold RPMI media (Cytiva) supplemented with 10% FBS (Gibco) and 1% PenStrep (Gibco) followed by incubation at 37 °C while rotating at 12 rpm for 30 min in an Incubated Rotator (Benchmark). The tissue was suspended in a digestion mix consisting of Dispase II at 1.25 mg/mL (Sigma), Collagenase II at 2 mg/mL (Gibco), and Collagenase IV at 2 mg/mL (Gibco). A 100 µM cell strainer (Falcon) was used to filter cells from the digested tissue. Dead cells were stained using Live/Dead Fixable Aqua Dead Cell Stain Kit (Invitrogen).

Cells were treated with Fc block (BioLegend) for 10 min before the addition of all the following antibodies to each sample: Brilliant Violet 605 anti-c-kit (BioLegend), APC-Cy7 anti-CD45 (BioLegend), FITC anti-FcεRIα (BioLegend) for the MrgprB2^+^ mice. For the X2^+^ mice, PE anti-X2 (BioLegend) was added. Data were collected using a FACSCelesta Flow Cytometer (BD) and analyzed using FlowJo (TreeStar). Mast cells were gated as live CD45^+^ c-Kit^+^ FcεRI^+^ and tdT^+^ (MrgprB2^+^ mice) or MRGPRX2^+^ (X2^+^ mice).

### Human mast cell culture

LAD2 (Laboratory of Allergic Diseases 2) human mast cells were cultured in StemPro-34 SFM medium (Life Technologies) supplemented with 2 mM l-glutamine (Fisher), 100 U/mL penicillin (Fisher), 50 µg per mL of streptomycin (Fisher) and 100 ng/mL recombinant human stem cell factor (Peprotech). The cell suspensions were seeded at a density of a million cells per mL and maintained at 37 °C and 5% CO_2_.

### Beta-hexosaminidase (β-hex) release assay (mast cell degranulation assay)

LAD2 X2 and LAD2 X2-KO cells were counted to 0.5 × 106 and resuspended in 0.4% BSA-HEPES buffer (HEPES buffer: HEPES 10 mM, NaCl 137 mM, KCl 2.7 mM, Na_2_HPO_4_.2H_2_O 0.4 mM, Glucose 5.6 mM, CaCl_2_.2H_2_O 1.8 mM, MgSO_4_.7H_2_O 1.3 mM, pH 7.4). Cells were then treated with varying doses of Compound 48/80 (Sigma), and PACAP-38 (Phoenix Pharmaceuticals) for 30 min at 37 °C and 5% CO_2_. Cells were centrifuged at 200 RCF for 5 min and the supernatant was added to a new plate. The remaining pellets were then lysed with 0.1% Triton X-100 (Sigma) in BSA-HEPES and added to a new plate. Supernatant and lysate were then treated with *p*-nitrophenyl *N*-acetyl-β-d-glucosamide (pNAG; Sigma-Aldrich) in 0.1 M sodium citrate buffer (pH 4.5) and incubated at 37 °C for 90 min. Finally, 0.4 M Glycine buffer (pH 10.7) was added. Plates were read at 405 nm and 595 nm to gather optical density, and β-hexosaminidase release was then calculated by the percent of total release.

### Mouse peritoneal mast cell isolation and calcium imaging

Adult mice were euthanized by CO_2_ asphyxiation and peritoneal mast cells were collected through a lavage of the abdomen^16^. Briefly, 2 × 6 mL of ice-cold mast cell dissociation medium (MCDM; HBSS (Gibco) with 3% FBS (Gibco) and 10 mM HEPES (Sigma), at pH 7.2) was injected into the peritoneal cavity and the abdomen was gently massaged for 60 s. The MCDM and cell suspension were carefully aspirated out of the abdomen and then centrifuged at 200*g* for 5 min at RT. The pellets were resuspended in 2 mL MCDM, layered over 4 mL of an isotonic 70% Percoll solution [850 µL MCDM, 40 µL HEPES, 30 µL 10× HBSS, 2.8 mL Percoll (Cytiva)], and centrifuged at 500*g* for 20 min at 4 °C. Mast cells recovered in the pellet were resuspended in DMEM with 10% FBS, 100 U/mL penicillin (Fisher), 50 μg/mL streptomycin (Fisher) and 25 ng/mL recombinant mouse stem cell factor (mSCF; Peprotech) and plated onto glass coverslips coated with 30 μg/mL fibronectin (Sigma). After 2 h of incubation at 37 °C and 5% CO_2_, mast cells were loaded with 1:2000 Fluo-4, acetomethoxy ester (Invitrogen) in Calcium imaging buffer (CIB: NaCl 125 mM, KCl 3 mM, CaCl2 2.5 mM, MgCl2 0.6 mM, HEPES 10 mM, glucose 20 mM, NaHCO3 1.2 mM, sucrose 20 mM, pH 7.4) for 30 min at 37 °C, followed by washing once in CIB. Fluorescence measurements were performed at 488 nm excitation.

### Generation of MRGPRX2 transgenic mice

The MRGPRX2 mouse line was generated by crossing the MrgprB2 Cre line^3,4,13,15^ to newly generated Rosa26-LoxP-STOP-LoxP-MRGPRX2 mice (Reverse Orientation Splice Acceptor, ROSA26^lsl-MRGPRX2^). The MrgprB2 Cre line was generated as detailed previously^16^. The progeny of these mice were then crossed to MrgprB2^−/−^ mice yielding mice in which only the MRGPRX2 transgene, and not the mouse MrgprB2, is expressed on mouse connective tissue mast cells (X2^+^ mice). As the MrgprB2 Cre promoter drives the MRGPRX2 expression in the MrgprB2^−/−^, mice that lack MrgprB2 Cre will not express MRGPRX2 (X2^−^) and served as control.

### Animal care and use

All experiments were performed with the protocols approved by the Animal Care and Use Committee of the University of Texas Medical Branch. All mice used were 6 to 12 weeks old. WT littermates were used in behavioral experiments with MrgprB2^−/−^ mice. The mice were housed in the vivarium with a 12-h light/dark cycle and all the behavioral tests were performed from 9 am to 2 pm in their light cycle. The housing group was five mice at maximum. All mice used were in the C57bl/6j mice background. A total of 137 mice were used. 98 mice for in vivo experiments, and 39 mice for in vitro experiments.

### Statistical analyses

All data are presented as means ± SEM. For experiments measuring facial mechanical hypersensitivity by counting the number of withdrawals from the filament out of 10 trials at multiple experimental time points, data were analyzed using a generalized linear mixed model (GLMM) with a logistic link function for binomial distribution and AR1 covariance structure for repeated measures (Group × Time in each sex and a sequential Sidak procedure for multiple comparisons between groups at each time point) (SPSS ver. 25, IBM). For analysis of β-hexosaminidase release from LAD2 mast cells, data were analyzed with a linear mixed model using plate as a random effect and genotype and drug condition as fixed effects, followed by pairwise comparisons on estimated marginal means, using the lmerTest and emmeans packages in R. For comparison of the number of mast cells responding, a two-tailed unpaired Student’s t-test was performed using GraphPad Prism.

### Ethics declaration

All experiments using animals conformed to the guidelines and regulations outlined in the National Institutes of Health Guide for the Care and Use of Laboratory Animals 8th edition. This study was conducted in accordance with Animal Research: Reporting of In Vivo Experiments Animal Research (ARRIVE) guidelines.

## Data Availability

All data supporting the findings of this study are available upon request to the lead contact author, Dustin P. Green at dugreen@utmb.edu. Any additional information required to re-analyze the data that is reported in this manuscript is available from the lead contact upon request, Dustin P. Green at dugreen@utmb.edu. This paper does not report the original code.
