# Author Correction: PACAP activates MRGPRX2 on meningeal mast cells to drive migraine-like pain

**DOI:** 10.1038/s41598-023-40309-z

**Published:** 2023-08-16

**Authors:** Sami Sbei, Taylor Moncrief, Nathachit Limjunyawong, Yaping Zeng, Dustin P. Green

**Affiliations:** 1 Institute for Translational Sciences, University of Texas Medical Branch, Galveston, TX USA; 2 Department of Neurobiology, University of Texas Medical Branch, Galveston, TX USA; 3 Center of Research Excellence in Allergy and Immunology, Research Department, Faculty of Medicine Siriraj Hospital, Mahidol University, Bangkok, 10700 Thailand

Correction to: *Scientific Reports* https://doi.org/10.1038/s41598-023-39571-y, published online 29 July 2023

The Acknowledgements section in the original version of this Article was incomplete.

“This study was conducted with the support of the Institute for Translational Sciences at the University of Texas Medical Branch, supported in part by a Clinical and Translational Science Award NRSA (TL1) Training Core (TL1TR001440) from the National Center for Advancing Translational Sciences, National Institutes of Health (Sami Sbei) and UTMB startup funding and a UT System Rising STARs award (Dustin P. Green). We would like to thank Dr. Jun-Ho La and Dr. Mark Baxter for their help with statistical analyses.”

now reads:

“This study was conducted with the support of the Institute for Translational Sciences at the University of Texas Medical Branch, supported in part by a Clinical and Translational Science Award NRSA (TL1) Training Core (TL1TR001440) from the National Center for Advancing Translational Sciences, National Institutes of Health (Sami Sbei) and UTMB startup funding and a UT System Rising STARs award (Dustin P. Green). We would like to thank Dr. Jun-Ho La and Dr. Mark Baxter for their help with statistical analyses. LAD2 cells were provided by the Metcalfe Lab at NIAID. MRGPRX2 mice were generated in Xinzhong Dong’s lab at Johns Hopkins.”

The original Article has been corrected.

